# ABO-identical versus incompatible platelet transfusion in patients with intracranial hemorrhage

**DOI:** 10.1371/journal.pone.0312602

**Published:** 2024-11-21

**Authors:** Lauren K. Dunn, Emily Venner, Matthew Nguyen, Jose Perdomo Trejo, Zachary Holley, Bhiken I. Naik, Jenna Khan, Michael Mazzeffi

**Affiliations:** 1 Department of Anesthesiology, University of Virginia Health System, Charlottesville, Virginia, United States of America; 2 Department of Neurological Surgery, University of Virginia Health System, Charlottesville, Virginia, United States of America; Colorado State University, UNITED STATES OF AMERICA

## Abstract

**Background:**

Patients with spontaneous and traumatic intracranial hemorrhage (ICH) are frequently transfused platelets to treat thrombocytopenia, platelet function defects, and reverse antiplatelet drugs. ABO-identical platelet transfusion has been suggested to lead to higher post-transfusion platelet increments compared to major-ABO incompatible transfusion. We hypothesized that patients who received ABO-identical transfusion would have higher post-transfusion platelet increments and superior neurologic outcomes.

**Methods:**

Adults with traumatic or non-traumatic ICH from January 1^st^ 2018 to December 31^st^ 2022 were identified using electronic medical records and international classification of disease (ICD)-10 codes. Patients were excluded if they lacked a platelet count within 24 hours before and within 24 hours after transfusion or if they received multiple platelet transfusions before their platelet count was remeasured. After stratification by ABO-identical, ABO-major incompatible, and ABO-minor incompatible transfusion, post transfusion increments were compared, as were clinical outcomes.

**Results:**

Among 167 patients who received platelet transfusion, 76 (45.5%) received ABO-identical transfusion, 54 (32.3%) received ABO-major incompatible transfusion, and 37 (22.2%) received ABO-minor incompatible transfusion. There were no significant differences in absolute platelet increment between groups. The median increment was 7x10^9^/L for ABO-identical platelets, 10x10^9^/L for ABO-major incompatible platelets, and 11x10^9^/L for ABO-minor incompatible platelets, *p* = .87. There was no significant difference in the percentage of patients discharged alive with modified Rankin score of 1 or 2 or cerebral performance category 1 or 2 between groups (*p* = .56 and .39 respectively). After adjusting for confounders in a general linear model there remained no associations between ABO compatibility and platelet increment after transfusion.

**Conclusions:**

Our data support similar efficacy for ABO-identical and ABO-incompatible platelet transfusion in patients with ICH.

## Introduction

Patients with spontaneous or traumatic intracranial hemorrhage (ICH) are frequently transfused platelets to treat thrombocytopenia, platelet function defects, and reverse antiplatelet drugs [1–3]. Notably, platelet transfusion remains controversial as a treatment for ICH and is not associated with improved outcomes in observational studies or randomized controlled trials, particularly when the indication for platelet transfusion is reversal of antiplatelet drugs [4,5]. In several studies, patients with ICH who were taking antiplatelet drugs had worse outcomes when they were transfused platelets [3,5].

The most recent guidelines from the American Heart Association/American Stroke Association published in 2022 do not recommend routine platelet transfusion in patients who are taking antiplatelet agents and do not exhibit thrombocytopenia and recommend that platelet transfusion only be considered in ICH patients being treated with antiplatelet agents to reduce the risk of postoperative bleeding [6]. In their most recent guidelines, the Association for the Advancement of Blood and Biotherapies (AABB) stated that they could not recommend for or against platelet transfusion in ICH patients who were taking antiplatelet drugs [7]. There were also no recommendations on whether transfused platelets should be ABO-identical, although this remains a controversial topic [8–10]. ABO-identical platelet transfusion has been associated with higher post transfusion platelet increments when compared against major-ABO incompatible transfusion (i.e. when recipient plasma has anti-A or -B antibodies that react with transfused platelets antigens) [9,11]. Nevertheless, there is no consistent evidence that transfusion of ABO-identical platelets leads to greater clinical efficacy, and hence the practice of transfusing ABO-incompatible platelets remains common in many hospitals to facilitate inventory management.

To our knowledge, there is a paucity of studies exploring whether ABO-identical platelet transfusion is superior in patients with ICH. In one small single-center observational cohort study, transfusion of major-ABO incompatible platelets was associated with reduced platelet increments, increased mortality (Odds ratio = 2.6), and worse modified Rankin scale at discharge in patients with ICH [12]. Although their findings were statistically significant, the authors acknowledged that their effect estimates had wide confidence intervals and were not precise. The aim of our study was to compare ABO-identical and ABO-incompatible (major and minor) platelet transfusion in patients with ICH. Our primary hypothesis was that patients who had ABO-identical transfusion would have higher post-transfusion platelet increments. Secondarily, we hypothesized that patients who had ABO-identical transfusion would have superior neurologic outcomes, as measured by cerebral performance category (CPC) and modified Rankin Scale (mRS) at the time of hospital discharge.

## Methods

### Study design

Our study was designed as a retrospective, observational cohort study and was approved by the University of Virginia Health Sciences Research Institutional Review Board (HSR-IRB# 24308). A waiver of informed consent was obtained and the study was exempt from ethics approval. The study was conducted following the guidelines outlined in the Strengthening the Reporting of Observational Studies in Epidemiology (STROBE) statement.

### Patients

Adult patients who experienced traumatic and non-traumatic ICH from January 1^st^ 2018 to December 31^st^ 2022 were identified in our electronic medical record (Epic Hyperspace, Verona, WI) using international classification of disease (ICD)-10 codes (parent codes I60, I61, I62, S06). Data was retrospectively collected between February 1, 2023 and March 31, 2024. The authors did not collect and record information that could identify individual participants during or after data collection. Patients were included in the study if they received a platelet transfusion during their hospitalization for treatment of ICH. Patients were excluded if they did not have a platelet count checked in the 24 hours before their platelet transfusion and also within 24 hours after transfusion. They were also excluded if they received multiple platelet transfusions before their platelet count was rechecked. Only a patient’s first platelet transfusion was assessed in the analysis if the patient received more than one platelet transfusion.

### Demographic and comorbidity data

For all patients, we collected demographic data, body mass index (BMI), and common comorbidities including diabetes mellitus, hypertension, prior stroke, coronary artery disease, chronic kidney disease, peripheral vascular disease, chronic obstructive pulmonary disease, and prior ICH. Laboratory data that were collected included hemoglobin, platelet count, international normalized ratio (INR), and activated partial thromboplastin time (aPTT). Data on anti-platelet drug use (e.g. aspirin or P2Y_12_ inhibitors) and neurosurgical procedures performed during hospitalization were also collected.

### Platelet transfusion data

Platelet transfusion was given at the discretion of individual providers, but general institutional guidelines are to transfuse platelets if patients have ICH and have received a P2Y_12_ inhibitor within 5 days of ICH or have a platelet count < 100,000x10^9^/L. All platelet units transfused in our institution are apheresis units. By the end of 2019, our institution had almost entirely transitioned entirely to pathogen reduced platelets in platelet additive solution (PAS). Pathogen reduced platelets were treated with the INTERCEPT ® system (Cerus, Concord, CA, USA), which uses amotosalen and ultraviolet A light to reduce bacterial contamination. All platelets were either irradiated or pathogen reduced.

Group O single donor platelets that were plasma incompatible with the recipient had isohemagglutinin titers <1:100 against the recipient’s ABO antigen(s), unless the component was a PAS unit. PAS replaces approximately 65% of plasma, therefore determination of titers is not routinely required for these units. Oldest platelets were selected first, then group-specific platelets whenever possible. Platelet transfusion was considered ABO-major incompatible when recipient plasma had anti-A or -B antibodies against transfused platelets antigens and was considered ABO-minor incompatible when plasma in the donor unit had anti-A or -B antibodies against recipient ABO antigens. There were 10 patients with bidirectional incompatibility (e.g. A platelet transfusion given to a B recipient), and all of these patients except one were in the ABO-major incompatible group.

### Study outcomes

The primary outcome was absolute post-transfusion platelet increment, which was calculated by subtracting the pre-transfusion platelet count from the post-transfusion platelet count. Secondary/exploratory outcomes were cerebral performance category (CPC) [13] and modified Rankin scale (mRS) [14] at the time of hospital discharge, as well as in-hospital mortality/hospice referral. Specifically, we reported the number of patients who were CPC ≤ 2, or mRS ≤ 2, because these categories reflect a good neurologic outcome.

### Statistical analysis

After stratification by transfusion type (ABO-identical, ABO-major incompatible, or ABO-minor incompatible) patient characteristics and outcomes were summarized as the median value and interquartile range or number and percentage of patients. Comparisons were made between groups using either the Kruskall-Wallis Test (continuous variables) or the Chi-squared categorical variables. To adjust for potential confounding variables, we fit a general linear model (proc GLM) where platelet increment was the dependent variable and independent variables included ABO compatibility as well as age, sex, surgical vs. medical patient, and BMI. The parameter estimates from the model represented mean differences in platelet increment along with 95% confidence intervals. For all statistical tests, p values < .05 were considered statistically significant. All analysis was performed using SAS 9.4 (SAS Corporation, Cary, NC, USA). The full analytic dataset is available as a Supplementary file.

## Results

**Fig 1:**
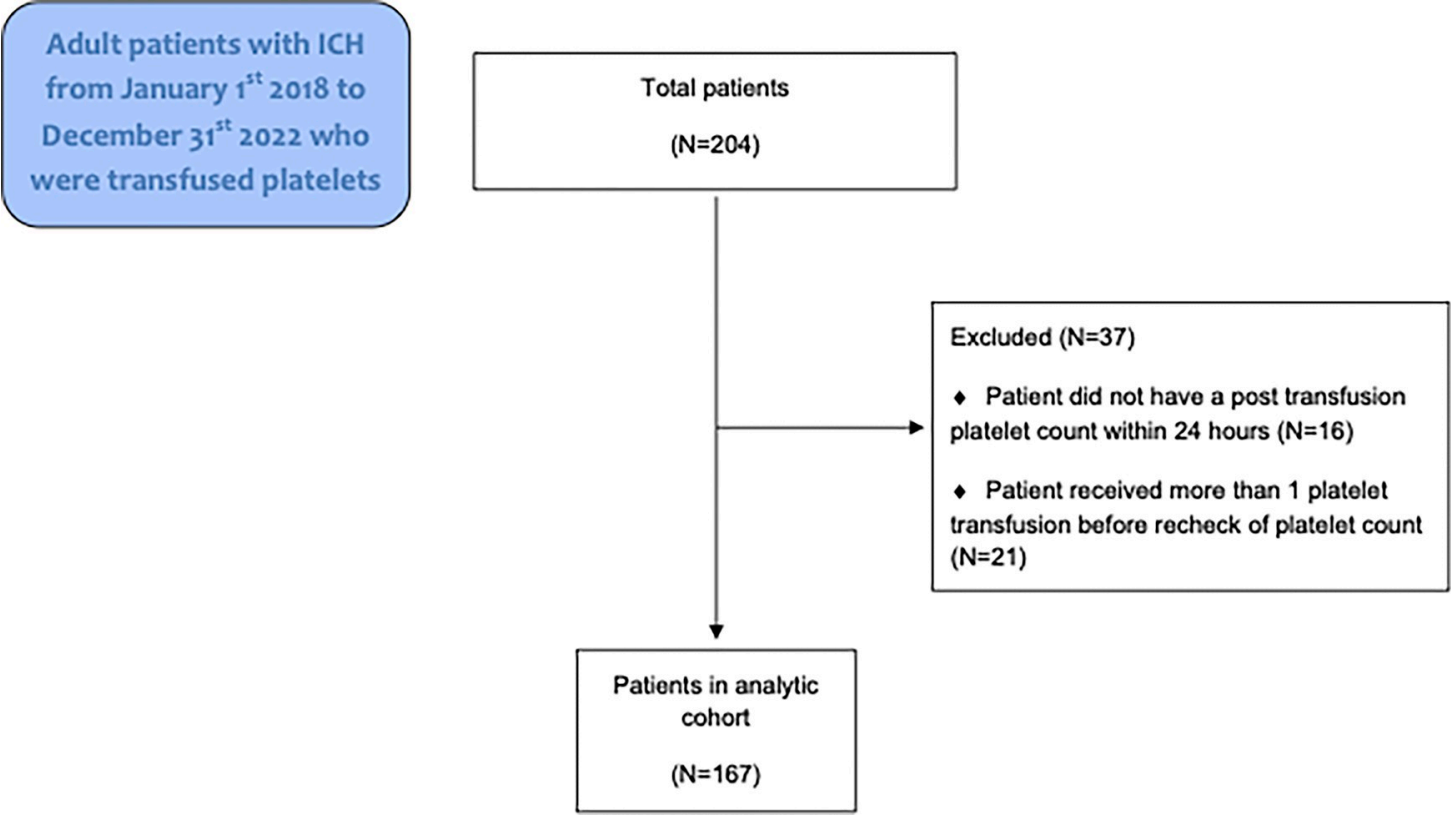
Consort diagram.

A total of 204 patients with ICH who received platelet transfusion were identified (Fig 1). Of the 37 patients excluded, 16 did not have a post-transfusion platelet count within 24 hours and 21 received more than one platelet transfusion. Among the 167 patients analyzed, 76 (45.5%) received ABO-identical transfusion, 54 (32.3%) received ABO-major incompatible transfusion, and 37 (22.2%) received ABO-minor incompatible transfusion. Pathogen reduction data were available for 75.4% of the platelet units that were transfused in the cohort, mainly platelets that were transfused after mid-2018 when pathogen reduction became more common in our institution. In the ABO-identical group 47.4% of platelet units were pathogen reduced, in the ABO-minor incompatible group 50.0% of platelets were pathogen reduced, and in the ABO-major incompatibility group 46.3% of platelets were pathogen reduced.

Patient characteristics, ICH type and characteristics, and platelet transfusion data are shown in Table 1. With the exception of male sex (*p* = .01) and body mass index (*p* = .03), there were no significant differences in patient characteristics between patients receiving ABO-identical, ABO-major incompatible and ABO-minor incompatible transfusions. Furthermore, no significant differences in type of ICH, trauma, intraventricular hemorrhage, location or procedural interventions (craniotomy or EVD placement) were observed between groups.

**Table 1:** Patient and ICH characteristics and platelet transfusion details.

Variable	ABO-identical N = 76	ABO-major incompatible N = 54	ABO-minor incompatible N = 37	*p* value
**Age**	66 [56, 75]	64 [53, 77]	63 [53, 72]	.89
**Male sex**	49 (64.5)	35 (64.8)	14 (37.8)	.01
**Race** **Black** **Am Indian** **Other** **No data** **White**	7 (9.2) 1 (1.3) 3 (4.0) 0 (0.0) 65 (85.5)	3 (5.4) 0 (0.0) 1 (1.9) 1 (1.9) 49 (90.7)	5 (13.5) 0 (0.0) 1 (2.7) 0 (0.0) 31 (83.8)	.71
**Body mass index**	25 [23, 29]	24 [20, 27]	25 [21, 28]	.03
**Diabetes mellitus**	18 (23.7)	12 (22.2)	8 (21.6)	.96
**Hypertension**	52 (68.4)	33 (61.1)	20 (54.1)	.32
**Prior cerebral vascular accident**	15 (19.7)	6 (11.1)	3 (8.1)	.18
**Prior ICH**	7 (9.2)	2 (3.7)	1 (2.7)	.27
**Coronary artery disease**	25 (32.9)	14 (25.9)	9 (24.3)	.54
**Chronic kidney disease**	15 (19.7)	5 (9.3)	3 (8.1)	.12
**Peripheral vascular disease**	3 (4.0)	2 (3.7)	3 (8.1)	.56
**COPD**	5 (6.6)	4 (7.4)	1 (2.7)	.62
**Hemoglobin (g/dL)**	11.8 [9.7, 13.7]	12.0 [9.9, 14.0]	11.8 [9.0, 13.1]	.61
**Pre-transfusion platelet count**	149 [75, 218]	112 [45, 218]	151 [74, 232]	.73
**INR**	1.2 [1.0, 1.4]	1.2 [1.0, 1.4]	1.1 [1.0, 1.4]	.94
**aPTT**	27 [24, 32]	27 [25, 30]	28 [27, 32]	.71
**Aspirin within 7 days**	42 (55.3)	33 (61.1)	20 (54.1)	.74
**P2Y**_**12**_ **within 5 days**	11 (14.5)	7 (13.0)	11 (13.5)	.97
**Statin use**	35 (46.1)	22 (40.7)	15 (40.5)	.78
**Type of ICH** **SAH only** **IPH only** **SDH only** **Multiple sites**	18 (23.7) 13 (17.1) 4 (5.2) 41 (54.0)	21 (38.9) 6 (11.1) 3 (5.6) 24 (44.4)	14 (37.8) 5 (13.5) 2 (5.4) 16 (43.3)	.60
**Traumatic injury**	25 (32.9)	18 (33.3)	8 (21.6)	.41
**Intraventricular hemorrhage**	27 (35.5)	16 (29.6)	17 (46.0)	.28
**Supratentorial ICH**	68 (89.5)	50 (92.6)	35 (94.6)	.62
**Craniotomy**	22 (29.0)	12 (22.2)	13 (35.1)	.40
**Had surgery during admission**	36 (47.4)	22 (40.7)	18 (48.7)	.69
**EVD placed**	14 (18.4)	11 (20.4)	9 (24.3)	.77
**Platelet transfusion during craniotomy**	10 (13.2)	8 (14.8)	6 (16.2)	.90
**Hours after transfusion post count checked**	4.0 [1.8, 7.3]	4.5 [0.75, 10.3]	4.5 [1.0, 10.0]	.82

aPTT = activated partial thromboplastin time, COPD = chronic obstructive pulmonary disease, EVD = external ventricular drain, ICH = intracranial hemorrhage, INR = international normalized ratio, SAH = subarachnoid hemorrhage, SDH = subdural hemorrhage, IPH = intraparenchymal hemorrhage.

Primary and secondary study outcomes are shown Table 2. There were no significant differences in platelet increment between groups. The median increment was 7x10^9^/L for ABO-identical platelets, 10x10^9^/L for ABO-major incompatible platelets, and 11x10^9^/L for ABO-minor incompatible platelets, *p* = .87. There was no significant difference in the percentage of patients discharged alive with mRS ≤ 2 or CPC ≤ 2 between groups (*p* = .56 and 0.39 respectively). There was also no difference in mortality or referral to hospice (*p* = .68)

**Table 2:** Study outcomes.

Variable	ABO-identical N = 76	ABO-major incompatible N = 54	ABO-minor incompatible N = 37	*p* value
**Absolute platelet increment (x10** ^**9**^ **/L)**	7 [–12, 24]	10 [–5, 21]	11 [–6, 26]	.87
**Modified Rankin score ≤ 2 at discharge**	17 (22.4)	16 (29.6)	11 (29.7)	.56
**CPC ≤ 2 at discharge**	24 (31.6)	23 (42.6)	15 (40.5)	.39
**In-hospital mortality or hospice referral**	29 (38.2)	19 (35.2)	11 (29.7)	.68

CPC = cerebral performance category.

Table 3 shows the results of the general linear model. There were no associations between major-ABO incompatibility and platelet increment (mean difference compared to identical, 5.81; 95% CI = -27.20 to 4.65) and minor-ABO incompatibility and platelet increment (mean difference compared to identical, 0.32; 95% CI = -19.5 to 20.15).

**Table 3:** General linear model results for platelet count increment with transfusion.

Independent Variable	Parameter estimate with 95% CI	p value
**Age (per year)**	-0.19 (-0.65 to 0.27)	.42
**Female sex vs. male**	-11.27 (-27.20 to 4.65)	.16
**Major vs. identical**	5.81 (-27.20 to 4.65)	.51
**Minor vs. identical**	0.32 (-19.5 to 20.15)	.97
**Surgical patient**	-10.5 (-26.21 to 5.13)	.19
**Body Mass index (per 1 unit increase)**	-0.29 (-1.59 to 1.00)	.65

*Parameter estimate represents estimated mean difference in platelet increment.

## Discussion

We compared patients with ICH who received ABO-compatible and ABO-major and ABO-minor incompatible platelets. No statistical differences in post-transfusion platelet increment or in secondary outcomes including mRS or CPC at discharge were observed between patients who receive ABO-compatible or ABO-incompatible transfusions. After controlling for potential confounders in a general linear model there remained no significant association between platelet ABO compatibility and post transfusion increment. These findings support the use of both ABO-identical and ABO-incompatible platelet transfusion in patients with ICH, as the two products appear to have similar efficacy and are associated with similar clinical outcome.

Our results contrast with the results of by Magid-Bernstein et al. who prospectively analyzed a cohort of 125 patients sequentially admitted with ICH to a single institution, 47 (38%) of whom received ABO-incompatible platelet transfusions [12]. ABO-incompatible transfusion was associated with lower absolute platelet count (2×10^3^cells per μL vs 15×10^3^cells per μL; adjusted coefficient β, −19; 95% confidence interval [CI], −35.55 to −4.44; *p* = .01), increased odds of mortality (adjusted odds ratio [OR], 2.59; 95% CI, 1.00–6.73; *p* = .05) and worse mRS (adjusted OR, 3.61; 95% CI, 0.97–13.42; *p* = .06) [12]. Slichter et al. similarly found that ABO-identical platelet transfusion was associated with higher post-transfusion increments [11]. Interestingly, Majid-Bernstein et al. did not observe significant differences in hematoma expansion between patients receiving ABO-incompatible vs ABO-identical platelet transfusions in their cohort [12]. They hypothesized that association between ABO-incompatible transfusions and poor outcome may be mediated by unmeasured confounders such as postexposure infections or thromboembolic events, but were unable to assess this due to their relatively small sample size. Of note, the platelets transfused in the study by Magid-Bernstein et al, were all suspended in plasma and therefore expected to contain higher levels of isohemagglutinins, soluble A and B antigens, and potentially other inflammatory molecules [12]. As the mechanism behind worse outcomes is not clear and these findings were not replicated in our study, it is possible that the difference in platelet product (pathogen reduced in PAS vs plasma) may be contributing to our results. Given the challenges associated with maintaining an adequate platelet inventory and the impracticality of providing only ABO identical platelets at many institutions, if pathogen reduction or PAS were confirmed as a mitigation strategy, this might provide a more feasible solution.

The 2016 PATCH trial demonstrated that platelet transfusion in patients on antiplatelet-therapy with spontaneous ICH was associated with increased 3-month mortality and risk of serious adverse event compared to standard care [5]. The results did not appear to be related to platelet preparation method or shelf life [15] and brought into question the routine transfusion of platelets in this patient population. The results of our study provide some small reassurance that outcomes for patients who do receive ABO-incompatible platelets are similar to those who receive ABO-compatible transfusions. Although 2022 guidelines from the American Heart Association/American Stroke Association do not recommend routine platelet transfusion in patients taking antiplatelet agents and who do not exhibit thrombocytopenia [6], there remains significant clinical equipoise regarding platelet transfusion in the setting of traumatic ICH [16]. There is a need for future prospective randomized controlled trials to investigate the role of platelet transfusion for patients with ICH being treated with antiplatelet agents undergoing surgical procedures to reduce the risk of postoperative bleeding.

Our study data show relatively small absolute platelet increments in ICH patients who received platelet transfusion. These small increments are consistent with prior studies of critically ill patients, where as many as 75% of patients have a poor response to platelet transfusion in terms of platelet increment [17]. Factors associated with small platelet increments include more severe illness, low pre-transfusion platelet count, and longer duration of platelet storage [17]. In a cohort study of 205 patients who received isolated platelet transfusion during surgery, median postoperative platelet count was 4x10^9^ lower than median preoperative count, suggesting that in surgical patients with bleeding increments are anticipated to be small or even negative in some cases [18]. In our cohort, a significant number of patients had surgery (approximately half), which may have impacted platelet increments.

Our study is limited by its retrospective design and relatively small sample size, Additional limitations are that only quantitative assessment of platelet number and not qualitative assessment of platelet function was included. Also, no radiographic evaluation of hematoma/bleeding expansion was performed, which is recommended in future prospective trials of hemostatic therapies [19]. We did not control for the potential impact of pathogen reduction on platelet increment or transfusion efficacy in our multivariable model because these data were not available for all platelet units in our electronic medical record review. Pathogen reduction has been suggested to reduce both platelet increment and efficacy and this may have biased our results to some degree [20–22]. For the platelet units that had pathogen reduction data available (75% of cohort) there was no difference in the percentage of platelet units that were pathogen reduced between ABO-identical and ABO-incompatible transfusions. Finally, for secondary outcomes, our sample size did not provide adequate power to compare rarer clinical outcomes such as transfusion-related thrombosis between groups.

In summary, in a single center observational cohort study of patients who received platelet transfusion for ICH we found comparable post transfusion increments and neurologic outcomes in patients who received ABO-identical and ABO-incompatible transfusion. Our data support current transfusion practice, but may also be underpowered to detect small differences in rarer clinical outcome such as thrombosis. Additional studies are needed to understand optimal platelet transfusion practices in patients with ICH.

## Supporting information

S1 Dataset (CSV)
